# Justice Is the Missing Link in One Health: Results of a Mixed Methods Study in an Urban City State

**DOI:** 10.1371/journal.pone.0170967

**Published:** 2017-01-27

**Authors:** Tamra Lysaght, Benjamin Capps, Michele Bailey, David Bickford, Richard Coker, Zohar Lederman, Sangeetha Watson, Paul Anantharajah Tambyah

**Affiliations:** 1 Centre for Biomedical Ethics, National University of Singapore, Singapore, Singapore; 2 Department of Bioethics, Dalhousie University, Halifax, Canada; 3 Department of Physiology, National University of Singapore, Singapore, Singapore; 4 Rimba, 4 Jalan 1/9D, Bandar Baru Bangi, Selangor, Malaysia; 5 London School of Hygiene and Tropical Medicine, London, United Kingdom; 6 Department of Medicine, National University Health System, Singapore, Singapore; International Nutrition Inc, UNITED STATES

## Abstract

**Background:**

One Health (OH) is an interdisciplinary collaborative approach to human and animal health that aims to break down conventional research and policy ‘silos’. OH has been used to develop strategies for zoonotic Emerging Infectious Diseases (EID). However, the ethical case for OH as an alternative to more traditional public health approaches is largely absent from the discourse. To study the ethics of OH, we examined perceptions of the human health and ecological priorities for the management of zoonotic EID in the Southeast Asia country of Singapore.

**Methods:**

We conducted a mixed methods study using a modified Delphi technique with a panel of 32 opinion leaders and 11 semi-structured interviews with a sub-set of those experts in Singapore. Panellists rated concepts of OH and priorities for zoonotic EID preparedness planning using a series of scenarios developed through the study. Interview data were examined qualitatively using thematic analysis.

**Findings:**

We found that panellists agreed that OH is a cross-disciplinary collaboration among the veterinary, medical, and ecological sciences, as well as relevant government agencies encompassing animal, human, and environmental health. Although human health was often framed as the most important priority in zoonotic EID planning, our qualitative analysis suggested that consideration of non-human animal health and welfare was also important for an effective and ethical response. The panellists also suggested that effective pandemic planning demands regional leadership and investment from wealthier countries to better enable international cooperation.

**Conclusion:**

We argue that EID planning under an OH approach would benefit greatly from an ethical ecological framework that accounts for justice in human, animal, and environmental health.

## Introduction

In the last 20 years, several novel zoonotic viruses with pandemic potential have emerged from Asia–Severe Acute Respiratory Syndrome (SARS), Nipah virus, and A H5N1, A H7N9 and other novel avian influenzas. Given the public health impacts of these pathogens, governments in the region and international agencies have been on high alert for zoonotic Emerging Infectious Diseases (EID) with endemic and pandemic potential. [1]

One Health (OH) is an approach to zoonotic EID management that calls for inter-disciplinary collaboration at the interface of human, animal, and environmental health. [2] The American Veterinary Medicine Association (AVMA) describes OH as the cross-disciplinary collaborative effort of researchers and policymakers working locally, nationally, and globally to “attain optimal health for people, animals, and our environment”. [3] Policies for responding to EIDs have been criticised for being too narrowly focused on public health aspects that affect humans without adequately accounting for non-human factors that contribute significantly to the emergence and threat of EIDs. [4] In this respect, OH has gained prominence in international policymaking discourses. [5–7] However, this lacks a rigorous, systematic articulation and defence of an ethical framework that grounds OH. In particular, the broader concept of justice is missing.

OH arguably calls for an ethical framework that fully appreciates the moral value of biodiversity and environmental health beyond their mere instrumental value to human health. [8] Despite widespread adoption of OH, existing approaches to zoonotic EID remain highly anthropocentric. They also tend to exclude responses that may be more effective, less costly, and potentially more justified from an ethical perspective than traditional approaches. [9] OH challenges conventional paradigms to re-orientate pandemic responses around wider community values, such as environmental health, [10] and take into account human as well as animal health. [11] In this respect, policy reforms are in need of ethical insight that might include elements of justice to address environmentally-linked health disparities. [12,13]

Justice is commonly conceived as fairness, relating to the fair distribution of resources and fair treatment of more or less equal stakeholders [14]. In bioethics, justice may be applied as a theoretical lens to explain observed norms and practices, and to guide complex decisions about rights, resource allocation and distribution, and burdens that are prominent in public health strategies. To explore the idea of an ethical framework that supports a more complete narrative for OH [15]–one that might include justice for both animals and humans–we conducted a mixed methods study of opinion leaders with expertise relevant to zoonotic diseases in Singapore.

### OH in singapore

Singapore is geographically and geopolitically important in the prevention and control of EIDs in Southeast Asia (SEA). [16] It is a small and highly urbanized country with few undisturbed, but highly diverse habitats where local flora and fauna are concentrated in small nature reserves. Singapore is a major importer of plants and animals, and a major transit hub for trade and tourism. [17] It is well-resourced with sophisticated public health and emergency response systems. [8] These systems were activated in 2003 to effectively contain the SARS outbreak. [17] From this experience, Singapore developed a multi-agency pandemic plan and infrastructure to respond to emerging infectious disease threats, which authorities activated during the outbreak of influenza A H1N1 in 2009. [18]

The primary planning assumption, based on the small agricultural and wildlife sectors in Singapore, is that “the first local human case is more likely to be imported from affected countries rather than developing from within Singapore through direct animal to human transmission”. [19] To help bridge knowledge gaps across disciplines and strengthen inter-sectoral cooperation, the three government agencies responsible for human health (Ministry of Health–MOH), food safety and animal and plant health (Agri-Food & Veterinary Authority of Singapore–AVA), and the environment (National Environmental Agency–NEA), initiated an OH platform in 2012. [20] This collaboration led to the development of contingency plans for the prevention and control of zoonotic EID, including multi-agency investigations in outbreaks of food-borne illnesses and surveillance of farms and animal premises done under the auspices of OH.

While there is a strong collaborative intent at the national policymaking level, we argue that even ostensibly OH policies too often adopt the goal of attaining *optimal health* for humans and only give limited consideration to health in non-human animals, and the impacts of public health strategies on the environment. For example, significant weight is often given to culling animals thought to harbour or transmit EID, even though this approach sometimes lacks a scientific evidence-base that demonstrates efficacy [21] and can damage important environmental and social niches, and community livelihoods. [22] This anthropocentric approach may be at least partly due to a limited scope for ethical deliberations. [3] To examine how ethics may apply to policymaking around zoonotic EID in Singapore, we engaged with opinion leaders in Singapore on the conceptual and ethical priorities of OH using a mixed methods approach.

## Methods

The Delphi survey is a systematic multistage method for obtaining, exchanging, and developing an informed opinion from a range of stakeholders to generate themes and agreement on a policy issue. [23] We modified our Delphi method from Holey et al (2007) [24] and combined the survey with qualitative interviews. The Institutional Review Board of the National University of Singapore approved the protocol (15 September 2014, A-14-174).

### Panel recruitment

We recruited panellists using purposive sampling and snowballing techniques to identify individuals with expertise in areas related to zoonotic diseases and their management. We sent an email advertisement to members of the local OH network, via the AVA, as well as to individuals identified from the investigators’ networks and websites of governmental regulatory agencies in Singapore. We sent potential panellists up to three emails and continued with up to three phone calls. Respondents were sent a formal Participant Information Sheet and a hyperlink to an online form where they consented to participating on the Delphi panel, and indicated their interest in being interviewed. Completion of the each survey implied their ongoing consent, and panellists who volunteered for an interview consented to the audio recording when commencing the interview. Those panellists who did not consent to the audio recording were excluded from the dataset. Records of the online consent forms and audio recordings are stored according to the University’s Research Data Management Policy. The IRB approved this consent procedure, including the waiver for written consent, on the basis that the target population of experts was non-vulnerable and the research involved no more than minimal risk.

### Survey method

We administered the Delphi survey to the panel over three successive rounds with results of each survey shaping the questions for the next. Each survey round was pilot tested and took less than 20 minutes to complete. We de-identified panellists and blinded their responses for analysis. Details on design and analysis of each survey round are provided in the supporting information (S1A Fig).

### Semi-structured interviews

We conducted six pre-survey and six post-survey semi-structured interviews. We excluded one pre-survey interview from the dataset, as the panellist did not consent to an audio recording. Thus, eleven interviews were audio recorded and transcribed for qualitative content and thematic analysis using NVivo software (QSR International, Doncaster, Australia). The project team initially identified content and themes for discussion in group meetings, which were further developed by two researchers (SW and TL) iteratively from the coded transcripts, and checked for consistency and agreement. The preliminary interviews were conducted to identify three scenarios and frame questions for Round 1 of the Delphi (supporting information S1A Fig). For the follow up interviews, preliminary results of the Delphi survey were shown to informants and discussed to help validate our findings. Interview guides for pre- and post-surveys are shown in the supporting information (S1D Fig).

## Results

Thirty-two experts consented to participate (see Table 1 for the expertise of panellists by employment sector). Of the 32 panellists recruited, 25 responded to the first and second survey rounds and 19 responded to the third round. This process produced a response rate of 78% and 59%, respectively. Analysis of the data was organised around three emergent themes: 1) conceptualisations of OH as promoting the health of humans, animals, and the environment; 2) the global and regional responsibilities of Singapore as an example of a high income country to monitor and contain EID in countries with fewer resources; and 3) the prioritisation of human and animal health in responding to EID. We discuss these themes in more detail with reference to bioethical concepts of justice below.

**Table 1:** Expertise of panellist by employment sector.

Panel’s Area of Expertise	Employment Sector	N
Infectious Disease	Medicine	4
	Academic	8
	Government	5
Veterinary Medicine	Private Practice	2
	Pathology	2
	Laboratory Animal Medicine and Science	5
	Zoological garden (Conservation)	2
	Industry	1
Environment and Wildlife Conservation	Environment	2
	Animal welfare	1

### Theme 1: OH as promoting the health of humans, animals, and the environment

From the responses in Round 2 to the conceptual questions (supporting information S1B Fig), analysis of the third and final Delphi survey round resulted in agreement with the statement:

“One health is the cross-disciplinary collaboration and communication between the veterinary, medical and ecological sciences and the relevant government agencies encompassing animal, human and environmental health policy and research.”

The single panellist who disagreed with this statement indicated in the open response a preference for the AVMA definition (see Introduction above), while another made the comment; “Would also like something to say what the purpose is—i.e. promotion and improvement of human/animal/environmental health”. When we discussed these findings in the post-survey interviews, panellists agreed that OH should not merely encourage collaboration but should have an overarching goal of *promoting* the health of humans *as well as* animals and the environment. They also agreed that attaining this goal would require regional and global cooperation.

### Theme 2: Global and regional responsibilities

The issue of Singapore’s role in the region emerged from the interviews. As indicated in this following statement, panellists we interviewed recognised the importance of supporting OH efforts beyond Singapore’s borders given the proximal risks of EID emerging within SEA:

“It’s going to involve global networks, particularly in countries where there is that cultural behavioural interaction. You want to go where people eat bats or kill primates, hunters; you want to go to those sorts of countries, because that’s the highest likelihood of those species jumps.” [Preliminary interview 2]

While noting the potential implications of EID on food security, human health, and tourism in Singapore, panellists also acknowledged the limited resources neighbouring countries had to effectively monitor and control the spread of EID, and expressed the need for Singapore to take on a greater leadership role and contribution to OH efforts in the region:

“Infectious diseases are not a local concern, but also a global concern. Yes so, you definitely need to work on a global scale… you definitely need to work globally, to be prepared for anything that may happen outside of your own country. And because of the fact that we import a lot of food and produce that means we also have to be very aware of what's happening around the region, and around the world, in terms of diseases. Not only for Singapore, but every other country as well” [Post-survey interview 2]

### Theme 3: Prioritising the health of human and non-human animals

The Delphi analyses in Round 2 (supporting information S1C Fig) indicated that when developing a plan of action, impacts on human and non-human animal health should have high priority, followed by the availability of manpower and healthcare resources, and economic impacts. However, when asked in Round 3 which of these priorities ranked the highest, most panellists agreed with the primacy of human health; with a range of different secondary priorities. From N = 19 responses, 16 ranked the impacts on human health as the highest priority, while 2^nd^, 3^rd^ and 4^th^ priorities were unevenly distributed across the other options. This outcome was informed by comments made in the interviews suggesting that these issues are inter-related and require careful balancing, as indicated in this statement:

“I think actually impacts on human health, economic and availability of manpower and healthcare resources are all inter-related, because if you develop a plan of action and you do not have the finances to do it you cannot carry it out. Even if you had prioritised human health in that response, and if you do not have the manpower and healthcare resources, you can’t carry that out as well. But if you prioritise the economic impacts then you would not do anything for anybody because you don’t want to spend the money on the human health, there really is so much balancing to do and it's not completely straightforward I guess. So therefore it's difficult to say ‘this is what you prioritise’.” [Post-survey interview 2].

The interviews also suggested that, although human health remained the priority, the panel was generally not supportive of culling healthy animal populations in response to EID, especially wildlife and companion animals:

“Apart from the public reaction and the fact that nobody really likes culling and killing the healthy animals […] you have to have quite a lot of manpower because, to do it effectively, you have to do it all at once. Otherwise it's never going to be effective.” [Post-survey interview 6].

Alternatives to culling, such as administering prophylactic drugs and vaccines to animals, were discussed. However, panellists recognised that the practicalities of these options depended on complex and multi-factorial considerations relating to the pathogen, route of transmission, animal host(s), and the availability of effective pharmaceutical measures and vaccines for both humans and non-human animals. No singular uniform policy option in response to EID emerged from our data.

## Discussion

Three key themes emerged from our analysis, which we will now discuss with reference to the published literature on OH and critically analyse through the conceptual lens of justice. This analysis forms the basis for our assertions about opportunities for developing OH ethics.

### Theme 1: OH as promoting the health of humans, animals, and the environment

We found agreement that OH approaches should promote the health of human and non-human animals, as well as the environment, through cross-disciplinary collaboration and communication between the veterinary, medical, and ecological sciences, and the relevant government agencies. This result is consistent with the AMVA definition of OH [3] as well as other similar approaches that emphasise inter-disciplinary collaborative efforts [4] and consideration of human and non-human indices of health. [8,11,15]

Panellists recognised the need to conserve biodiversity even in urban contexts. Heavily disturbed habitats, such as green belts and nature areas in and around cities, may pose a risk for zoonotic disease emergence since urban biodiversity promotes more direct and indirect (e.g., animal excrement) contact between humans and non-human animals. [25] Urban centers also create conditions for close contact between people and companion animals–living in large, close knit, shared dwellings and environments–and imported farm animals at slaughterhouses and live animal markets. These places raise specific risks because emergent or imported pathogens can become established and hide in exotic and native fauna that become extraordinarily difficult to control in any spill-over event. Promoting the conservation of natural areas in peri-urban contexts contributes to wellbeing and aesthetics, and in other contexts, greater biodiversity may benefit humans as more species-rich ecosystems can buffer against EID. Although not conclusively demonstrated in urban conditions, these buffering capacities and ecosystem services (that, for example, provide clean air and water, fertile soils, food, pest control etc.) are much more likely to be viable in species-rich and intact ecosystems. [26] As ecosystems lose biodiversity, they become less functional and are more likely to lead to the emergence of zoonotic EID.

### Theme 2: Global and regional responsibilities

Panellists suggested that countries that can (via capacity, expertise and resources), should take on greater international responsibilities and leadership roles in managing EIDs, especially those with potential global health impacts. Despite the risks of a zoonotic disease emerging from local animal populations, the panel shared the view of regulatory agencies that zoonotic EIDs with pandemic potential were more likely to emerge from neighbouring countries and enter into Singapore through one of its numerous entry points. [19] Thus, enlightened self-interest to protect the national population may partially be the reason for Singapore to support efforts beyond its borders. It is striking, however, that the panel recognized the importance of outreach beyond Singapore in the control of zoonotic EID.

As noted previously, Singapore is regionally situated within the heart of a region likely to be the epicentre for emerging and re-emerging infectious diseases. [1] In a Raffles Dialogue published in *Lancet Global Health*, several eminent and influential commentators suggested “we must realise that we live in a small and interdependent ‘global village’, where Asian countries need to assume greater leadership of our global village councils”. [27] They also note that Asian states have “shown little inclination” to take on more international responsibilities despite gaining global economic power in recent decades. This lack of global citizenship is reflected in Singapore’s contribution to the Ebola control efforts, which was similar to countries like Spain and Luxembourg but far lower than the economically comparable countries of Norway, Denmark and Kuwait, which have tended to adopt a more international approach to global health aid. [28]

Financial contributions are not the only way that countries promoting OH can support their neighbours and the wider global community. The Ministry of Foreign Affairs runs the Singapore Cooperation Program, which has provided training and education for more than 100,000 government officials from 170 developing countries in the last 25 years. [29] Despite these efforts, panellists felt that it would be challenging for neighbouring countries, where Singapore sources most of its food, to detect and contain EID. While the responsibilities that high income countries have towards communities that lack resources to control EID effectively have been discussed previously, [27] the relationship between rural populations and urban centres, where there is limited undisturbed fauna and flora, is under-recognised in many discussions about applying OH to pandemic planning.

### Theme 3: Prioritising the health of human and non-human animals

The panel recognised the issue of culling animals as highly controversial and extremely difficult to implement effectively within an urbanised area. In particular, the panel noted the potential public objection to the culling of healthy animals, including pets, and the logistics of quickly containing and killing large numbers of animals. This finding challenges plans that prioritise culling animals as an immediate response to an emergent threat. [30]

Singapore has conducted culling exercises with poultry farm chickens in preparation for an avian influenza outbreak. [31] While controversy around these exercises was less apparent, there is resistance towards culling other animals, such as the native Macaque monkeys, to reduce health risks to human populations. [32,33] Of note, community outrage ensued at the 2003 culling of stray feline populations when there was a misguided concern that domestic cats (not related to civet cats who are hosts for SARS) were sources for the infection. [34]

Indeed, the effectiveness of culling domestic and wild animal populations is increasingly coming under scrutiny following the results of a randomised trial in the UK of badger culling that showed the practice to be ineffective at reducing disease transmission. [21] Furthermore, evidence suggests that culling may paradoxically increase disease risk in humans and animals by encouraging spill-over. [35] A reduction in natural hosts can force the pathogen or vector to seek another animal host which could be even more hazardous for humans and other animals. Alternatives, such as animal vaccinations, have been proposed, and proof of principle was demonstrated with the development of a vaccine against the Hendra virus for horses during the 2012 outbreak in Australia. [36,37] However, existing frameworks used to justify the time, costs, and practicalities of implementing these and other alternatives, currently lack ethical basis for considering both impacts on human communities as well as non-human animal populations.

## Justice as a Lens for OH Policymaking

Our findings, as categorised under the three themes, may be viewed through the conceptual lens of justice. The Bioethics Advisory Committee of Singapore has interpreted the concept of justice as meaning: “access to the benefits of research, and the burden of supporting it, should be equitably shared in society.” [38] More generally is the idea that societies should organise themselves to secure cooperative benefit from and for its members; and justice provides a set of principles to allocate the benefits and burdens of this cooperation fairly (i.e. procedural justice). The notable 20^th^ Century philosopher, John Rawls, states: “social cooperation makes possible a better life for all than any would have if each were to live solely by his own efforts”. [14] This concept resonates with many ideas of public health, including the influential UK Royal College of Physicians’ statement that public health is:

‘The science and art of preventing disease, prolonging life and promoting health through organized efforts of society’. [39]

Although the basic idea of justice has been extensively developed in philosophical writing, and is often applied to public health frameworks, the bioethical discourse around OH has failed to resolve conflicts arising between the interests and values identified in this study–that is, human vs non-human health, and local vs regional and global responsibilities. That there might be competing claims between humans and non-human animals is missing from the current OH framework.

Even when OH accounts allude to these conflicts, they do not often indicate what is, or who counts as, a member of society. Thus, they do little to address the conflicts around the fair distribution of benefits and burdens. We suspect that, more often than not, anthropocentric accounts of justice–focussed on fair distribution of benefits of burdens within an essentially human community–are being favoured over more inclusive approaches. Inevitably, humans become the focus of attention, while animals are subsumed under welfare terminology that includes positive conditions for their care in production farming, experimentation and confinement, but not obligations to their interests. Justice addresses the problem of fairness when there are competing claims to resources, or benefits to enjoy and burdens to shoulder. Therefore, we suggest that OH ethics must include non-human animals and environmental health within a *just* conception of health. In doing so, OH broadens the ethical discourse to develop strategies that include zoological and ecological concerns.

An OH lens of justice should also encourage urban states to assume greater responsibility as global citizens. This *strengthens* ideas of global justice by challenging the self-interests of cities (and city-states). Despite having (sometimes) little landmass and few links to wilderness, they provide localised niches for EID and become hubs for the rapid spread of diseases; they also have considerable resources situated in their infrastructure and unique demographics (e.g. a concentration of skilled professions and political decision making). In this respect, Laurie Garrett has shown how governments worldwide have ‘declined to even entertain an increase in annual assessment rates to fund World Health Organisation for nearly four decades’. [40] This underfunding of shared resources had led users to act in a self-interested and independent manner, which affects wealthy countries occasionally but affects lower income rural settings on a daily basis in not providing adequate human and animal health care.

Applied to our study, this conceptualisation of justice should also discourage policies that apply disproportionate burdens on animal populations. Instead, they should encourage interventions that benefit both human and non-human animal populations. Evidence suggests that such approaches might be more effective to ensure the health of all stakeholders than current planning assumes. [22] From this perspective, the retention of ineffective and potentially harmful responses, such as culling, is not ethically sustainable partly because they unfairly lay the burden of shared diseases with humans onto animal populations. [9] Culling also often exerts emotional and economic burdens on the agriculture sector and, therefore, unfairly distributes the harms to rural communities while benefitting those in business and tourism primarily in urban centers. Hence, planning should prioritise alternatives that have the potential to benefit *both* rural and urban communities, such as vaccines, better husbandry practices, and land management.

## Conclusion

Our study examined the conceptual and ethical priorities of OH for preventing and managing EID in Singapore. We used a modified Delphi survey and qualitative interviews with a panel of opinion leaders in Singapore, and had a good response from a diverse range of experts. However, there may have been a selection bias with those having a greater interest in OH being more likely to respond to the surveys. Additionally, since the study focussed on Singapore within tropical SEA, some of our findings may not be generalizable to all countries. Nevertheless, our findings may be informative for policymaking in other urbanised states.

While principles of justice have not been widely recognised as an important component of zoonotic EID planning, there is support among the OH community for such an approach. Our interpretation of the emergent themes suggests that opinion leaders in a major urban center would recognize the importance of justice in dealing with the less well-off communities and the non-human inhabitants of our ecosystems. While ethics has taken a greater role in the OH approach to pandemic planning, in particular, research ethics and questions about novel therapies in vulnerable populations, our findings suggest that more work needs to be done on the role of justice in preparation for zoonotic EIDs and respect for the environment and non-human animals. We have argued that it is insufficient for an OH framework to merely subsume ideas about public health and animal welfare without accounting for the inevitable conflicts that arise between diverse interests and the obligations that are owed to all who share the burden of disease. In other words, OH needs to do more rather than merely assimilate, uncritically, the problematic balancing of interests found in the rights-based discourse of public health ethics. This critical area is in need of further development to ensure that the OH approach is as holistic and comprehensive as possible for the benefit of all.

## Supporting Information

S1 Fig ASurvey Methods and Analysis.BResponses to Conceptual Question.CResponse to Priorities Question.DInterview Question Guides.
(DOC)
