# Supplementary material for: Justice Is the Missing Link in One Health: Results of a Mixed Methods Study in an Urban City State
Source: PLoS One. 2017 Jan 27;12(1):e0170967. doi: 10.1371/journal.pone.0170967 (PMC5271361; doi:10.1371/journal.pone.0170967)
Supplement: S1 Fig — ASurvey Methods and Analysis.BResponses to Conceptual Question.CResponse to Priorities Question.DInterview Question Guides. (DOC) [file pone.0170967.s001.doc]

# Supplementary Materials

## Fig A. Survey Methods and Analysis

Round 1: Panellists were asked if they had heard about OH before; the one panellist who had not, was given the AVMA description. They were then presented with open-ended questions in response to one of three randomly assigned scenarios:

1. A group of dead birds are found in the Sungei Buloh Wetland Reserve with 10 relatively fresh carcasses. Many Singaporeans are also ill with flu-like symptoms, with a higher than usual rate of people turning up at the polyclinics with upper respiratory symptoms. The hospitals have also noticed a spike in pneumonia cases. There is a proposal to quarantine all visitors to the Sungei Buloh area since the flu-like cases began, but that will be difficult as there is no entry charge into the reserve to track visitors and some visitors from overseas may have already left Singapore. There are also proposals to attempt to control migratory birds who visit the reserve.
2. A cache of young dogs intended for domestic ownership is found on small fishing boat during a routine inspection. The boat had come from Sumatra. The dogs are taken to Sembawang Animal Quarantine Station where one is showing signs of rabies. On further investigation, it turns out that the boat had done a number of such trips and that dogs have already been delivered to customers in Singapore.
3. It is coming up to Chinese New Year. During a routine pest investigation of a Hawker Centre in China Town, rats are found to be using the crawl space near the roof. The roof space links a number of restaurants and food courts. The rats are tested and found to be carrying Hantavirus. Some owners and contract cleaners have severe respiratory problems.

Responses were recorded and analysed qualitatively to identify themes and extract statements presented to the panel in the next round.

Round 2: For the conceptual questions, panellists were asked to agree or disagree with a list of statements describing OH using a Likert scale (1 = strongly agree, 5 = strongly disagree) and to indicate which statement most closely reflected their own views. For the priority questions, panellists were asked to choose up to three options from a list that should be prioritised when developing a plan of action in response to a possible EID.

Round 3: For the conceptual question, a single statement was constructed from panel responses in Round 2. As shown in Figure 1, the items with the highest frequencies and means < 2 (indicating agreement) were: B) The integration of animal, human and environmental health policy and research (
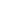
=1.62); A) The inter-relationship between animal, human and environmental health (
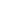
=1.83); and D) The cross-disciplinary collaboration and communication between the veterinary, medical, and ecological sciences and the relevant government agencies (
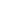
=1.83).

## Fig B. Responses to Conceptual Question.

Question asked:“Indicate whether you agree or disagree with the following statements (listed from A-H) about One Health. Respondents (n=25) indicated their agreement on a Likert scale of 1-5 (1 = strongly agree, 5 = strongly disagree)”


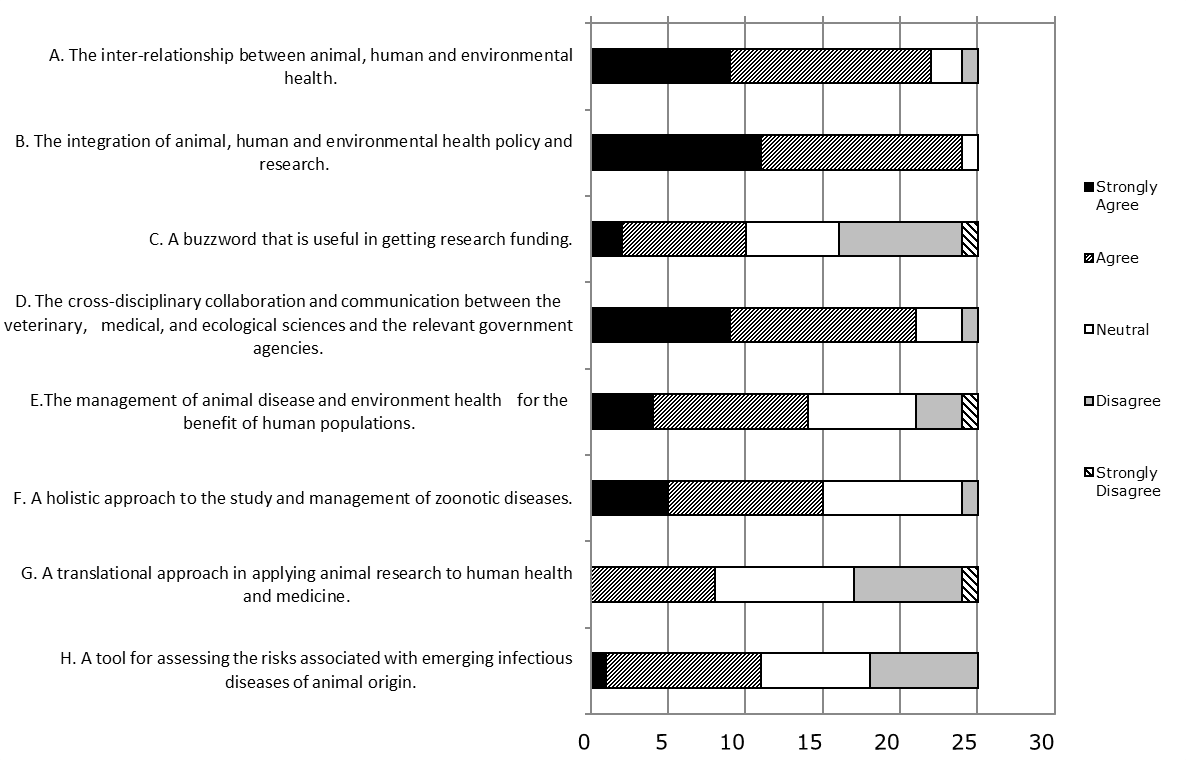


These items were collapsed into the following statement, which panellists were asked to agree or disagree with of a four-point Likert scale (1 = strongly agree, 4 = strongly disagree), and to indicate how it could be improved in an open-ended question:

One health is the cross-disciplinary collaboration and communication between the veterinary, medical and ecological sciences and the relevant government agencies encompassing animal, human and environmental health policy and research.

For the priorities question, the items with most number of responses in Round 2 were presented to the panel again in Round 3 to determine if any should be prioritised over the other when developing a plan of action. As shown in Figure 2, the items with the highest frequencies (>7) were: Impacts on human health (n=21); Welfare and health of animals (n=12); Availability of human and health resources (n=10); Financial costs of implementing the plan (n=7), and; Economic impacts on individuals, business and the government (n=7).

## Fig C. Response to Priorities Question.

Question asked: “When developing a plan of action, which of the following should be prioritised?” Respondents (n=25) could choose up to three options. The most frequent responses were selected for inclusion in Round 3.


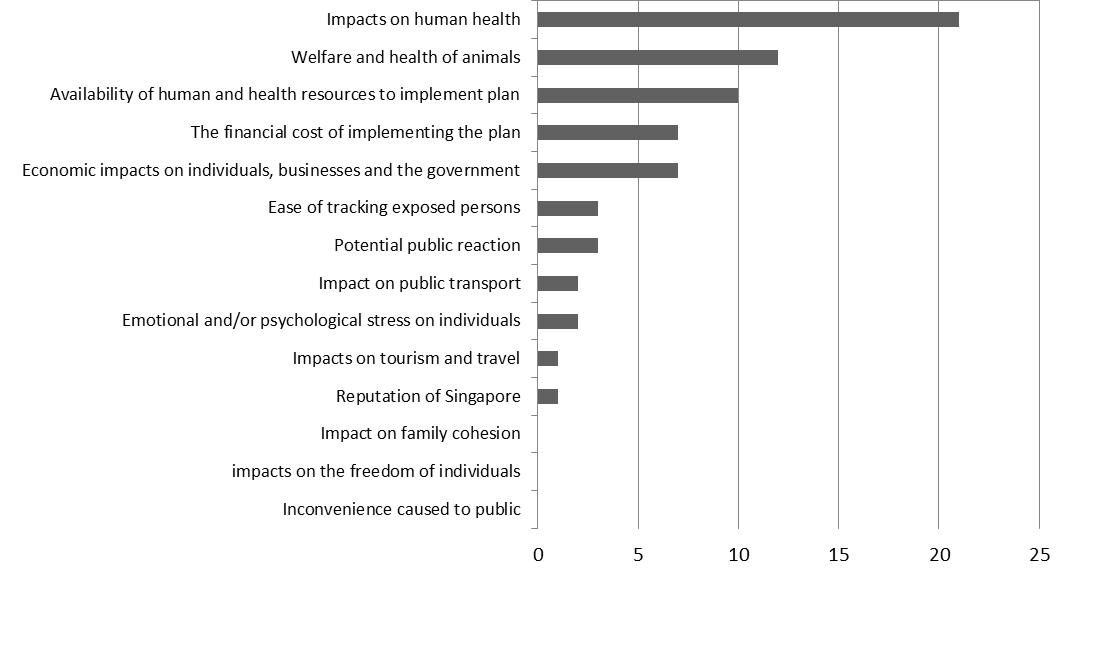


The wording of the second item was amended to be consistent with the first, and the third for clarity, while the last two were collapsed into a single item of economic impacts. Panellists were asked to rank in order of 1-4 (1 = highest priority), which of the following considerations should be prioritised in developing a plan of action.

- Impacts on human health
- Impacts on animal health or welfare
- Availability of manpower and healthcare resources
- Economic impacts

## Fig D. Interview Question Guides.

The questions in the interview guides are only indicative of those asked of the panellists. Unlike a structured survey, qualitative semi-structured interviews allow researchers to respond, follow up and probe according to participant responses. Thus, specific questions to individual panellists interviewed differed accordingly.

## Pre-survey interviews

*Pathogen risks*

1. In your opinion, what are the most important infectious diseases potentially threatening the health of humans and non-human animals in Singapore?
2. Of these, which pose the main threat in terms of human mortality and/or morbidity?
3. Which pose the main threat in terms of animal mortality and/or morbidity?
4. Which pathogens pose the main threat in terms of economic burden (both direct and indirect)?
5. Which pathogens would be most amenable to preventative measures?

*Risk populations*

1. In your opinion, which human populations in Singapore are most at risk in case of a disease epidemic?
2. What could be done in order to reduce that risk? What do you know is being done? What more could be done?
3. What are the animal populations in Singapore that are most at risk in case of an infectious disease outbreak?
4. What could be done in order to reduce that risk? What do you know is being done? What more could be done?

## Post-survey interview guide

*Conceptualisation of OH*

1. What do they mean by ‘inter’ and ‘holistic’, how do they differ and which best reflects OH
2. Do you think OH is just a buzzword concept – is OH really just a buzzword or does it hold substance?
3. The discrepancy between responses to questions 1a and 1b in Round 2.. Is it because 1a reflects their professional view of what the right definition of OH *is* while 1b is more of what they personally think it *should* be (or means to them)?
4. Should the definition include a purpose for OH? Should OH research be integrated with public health policy – i.e. targeting research that leads to policy and/or improved health outcomes? How can the impacts of OH be monitored?
5. Someone suggested that the AVMA definition is preferable.. this definition includes a component of research working at global/regional/local levels..
   1. How important is it for the OH definition to include this and is it practical to make OH work across transnational and geopolitical boundaries? Should the definition be aspirational or normative (i.e. a rule), or one that can be practically operationalized?
   2. Should Singapore (and other wealthy countries) direct research efforts to countries/areas where novel zoonoses are likely to emerge? And should Singapore take on a greater leadership role in the region? Is ASEAN a good model? What are the political practicalities?

*Framing of OH*

1. Were the questions and responses in the Delphi too anthropocentric?
   1. Was there sufficient opportunity to bring out ecological or environmental responses or was it too focussed on human actions and interests?
   2. Why would human interests dominate the discourse and what practical implications does this focus have for a framework that is supposed to situated at an interface that is not human centric, but also includes animal and ecological concerns.
2. Is this focus on humans merely a pragmatic way of framing the discussion to promote policy action, or is there genuine belief that human interests should always be the highest priority? Do you believe that human health/interests are the top priority?
   1. Ultimately, are the moral questions all just about human interests? Do the interests of animals and/or environment ever prevail without reference to human interests? Do we look after the health of animals and ecosystems (creating a false dichotomy) because that is how human health benefits (and vice versa)
   2. If non-human species are to be protected and benefit from OH policies; which ones count and how should that determined?
3. Is culling generally viewed as an unviable option, or at least one that is not considered an immediate priority in response to an emerging threat?
   1. What counts as evidence when establishing the effectiveness of vaccines and culling in reducing the risk of disease transmission?
4. How was ‘economic impacts’ understood:
   1. Do impacts include availability of manpower to respond? Do impacts include availability of people with appropriate expertise to respond?
   2. Were responses also affected by how panellists viewed our agri-industry as being small, with tourism being the biggest?
   3. In terms of raw costs of implementing the plan, or from loss of revenue via reduced tourism/transit etc;
   4. Who is impacted (government, corporations or small businesses); impacts on small businesses or on the country/government; and on what industries? Did this influence the distribution of responses in Q3R3?
5. Were panellists responding to the questions as “what you think should be done, or what the government thinks should be done?” How should decision makers prioritise these impacts if at all?
